# Osteolytic Bone Lesions – A Rare Presentation of AML M6

**DOI:** 10.4084/MJHID.2015.017

**Published:** 2015-02-15

**Authors:** N. Geetha, K.P. Sreelesh, M. J. Priya, V.S. Lali, N. Rekha

**Affiliations:** 1 Department of Medical Oncology. Regional Cancer Centre. Trivandrum 695011, India; 2 Department of Pathology. Regional Cancer Centre. Trivandrum 695011, India

## Abstract

Acute myeloid leukemia (AML) M6 is a rare form of AML accounting for < 5 % of all AML. Extramedullary involvement is very rarely seen in this entity. Skeletal lesion has not been described in AML M6 before. We discuss the case of a 17 year old boy with AML M6, who presented with osteolytic lesion of right humerus. He was treated with induction and consolidation chemotherapy. The present case is the first report in literature of AML M6 presenting with skeletal lesions.

## Introduction

Acute erythroid leukemia or Acute myeloid leukemia (AML) M6 is a rare form of AML. It accounts for < 5 % of all AML.^1^ AML M6 is otherwise known as Di Gugliemo syndrome, and it is a disease of adults. Extramedullary involvement is very rarely seen in this entity, and bone involvement is extremely rare. We present the case of a 17 year old boy with AML M6, who presented with predominant skeletal disease.

## Case Report

**Figure 1:**
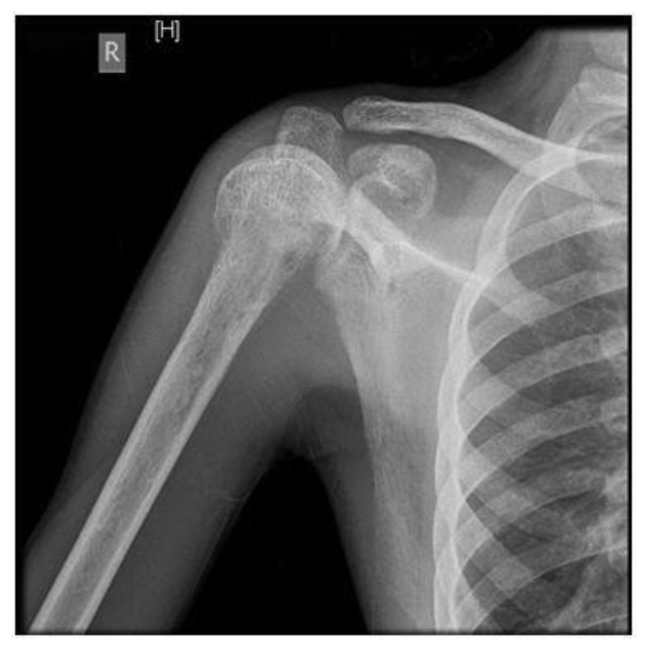
Xray right shoulder AP view showing irregular permeative type of lytic lesions involving proximal metadiaphysis region of the right humerus, cortical breaks, interrupted periosteal reaction and a wide zone of transition. No significant soft tissue component is present.

A 17 year old boy presented with progressively increasing pain in right shoulder since 6 months, pain in right chest wall and gluteal region since 3 months. He gave history of intermittent fever and general weakness. A radiograph of right shoulder showed an irregular permeative type of lytic lesion involving proximal metadiaphyseal region of right humerus. Cortical breaks and interrupted periosteal reactions were present (Figure 1). He had undergone a biopsy from the humeral lesion prior to presenting to us.

**Figure 2:**
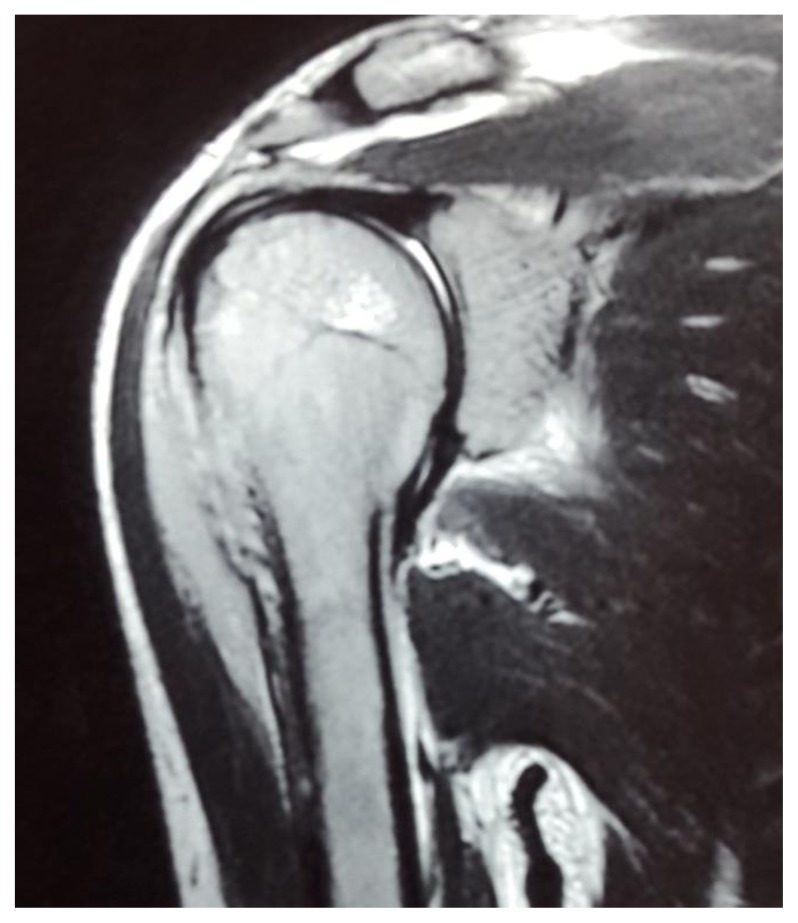
Magnetic resonance imaging showed focal cortical lytic lesion in the head of right humerus and greater tuberocity

**Figure 3:**
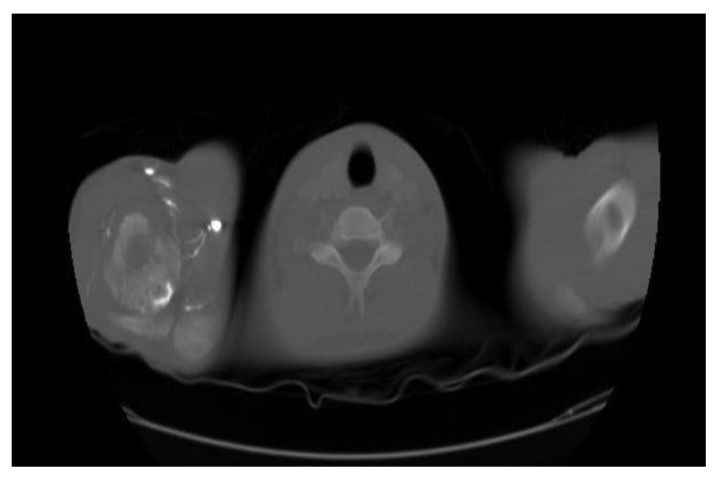
CT both shoulder axial view (bone window) showing irregular destructive lytic lesions of right upper humerus.

Examination showed a sick boy with a performance status of 4, he had pallor, tenderness of right shoulder and hepatomegaly. His hemoglobin was 7.4gm%, total leucocyte count 3800/mm^3^, platelet was 1,67,000/mm^3^ and peripheral smear showed 6% abnormal cells. Serum chemistries were normal, and LDH was 532 IU/L (Normal 313–618U/L). Magnetic resonance imaging showed focal cortical lytic lesion in the head of right humerus and greater tuberocity, glenoid and corocoid process and right clavicle with moderate periosteal reaction (Figure 2). A computed tomogram showed permeative destruction of both shoulder joints and pelvic bones (Figure 3).

**Figure 4:**
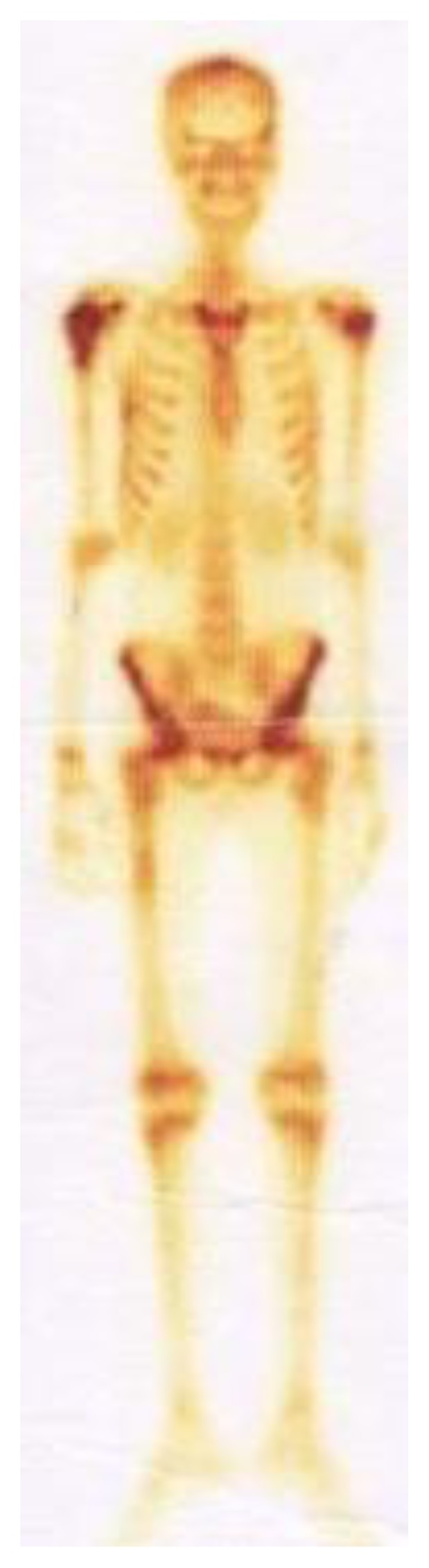
Bone scan showing increased uptake over both humerii, trochanters and shaft of right femur

**Figure 5:**
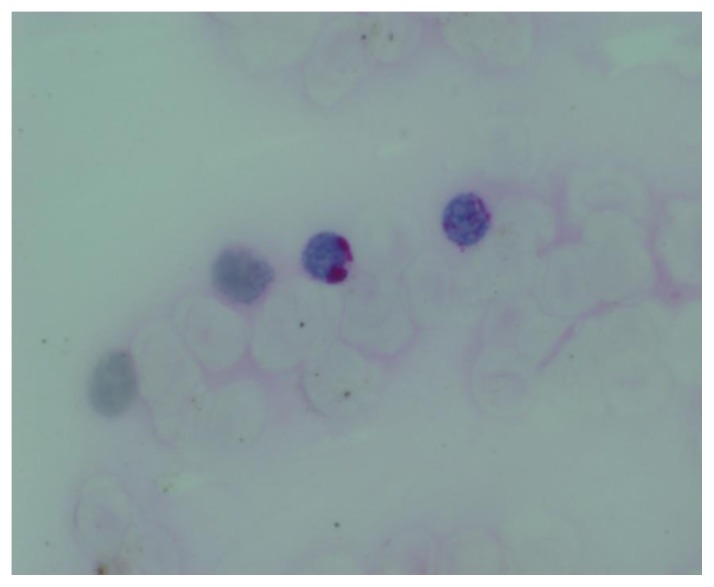
PAS X1000. Blasts show PAS block positivity

**Figure 6:**
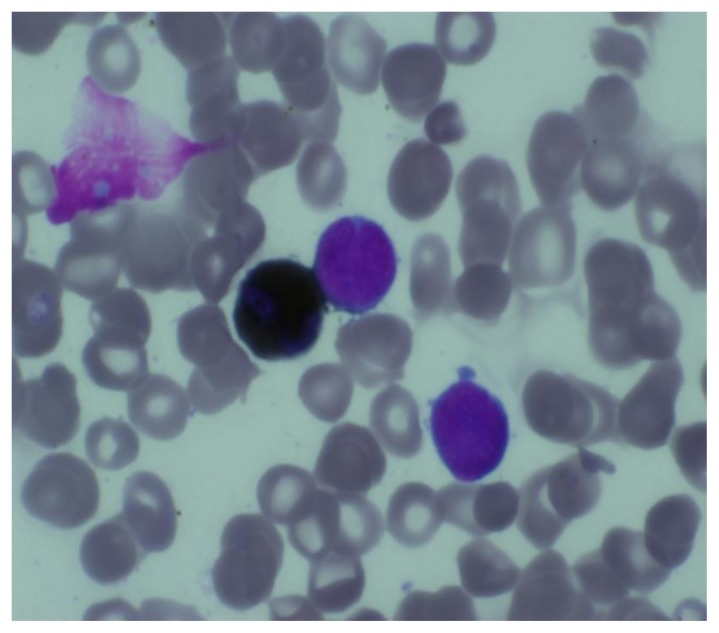
Myeloperoxidase x 1000. Blasts are myeloperoxidase negative

**Figure 7:**
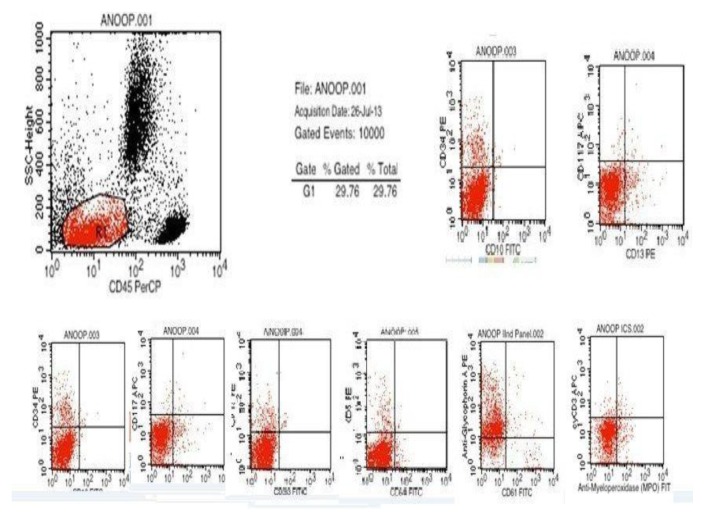
Flowcytometry dot plot scan showing blast cells with negative uptake for CD13,CD33,CD34,CD68,CD10,CD117,MPO and positive uptake for anti glycophorin A (70%)

**Figure 8:**
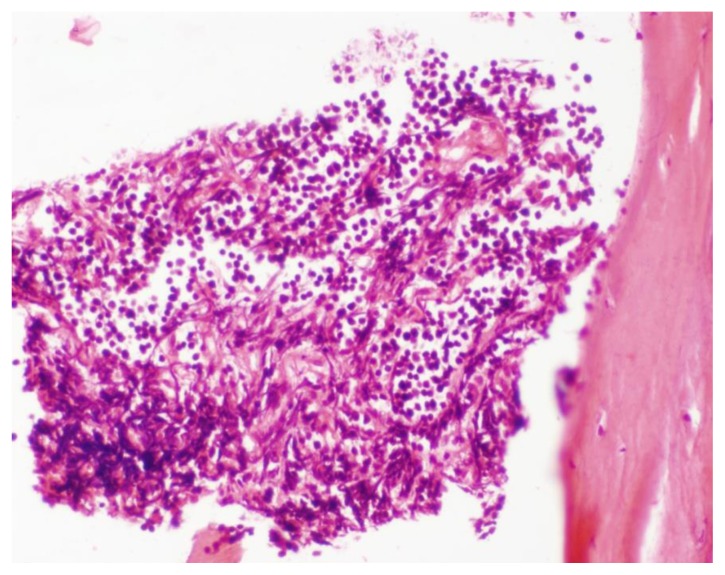
H&E x 400. Section from bone shows infiltration by blasts.

**Figure 9:**
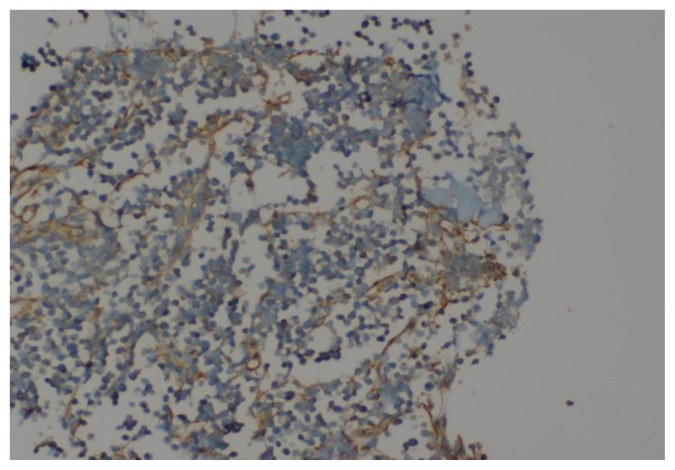
IHC analysis for LCA showing negative uptake by blast cells

**Figure 10:**
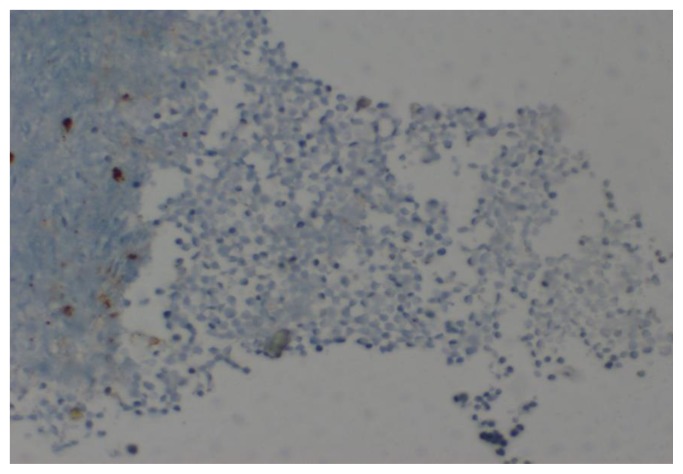
IHC analysis for mic 2 showing negative uptake by blast cells

A Tc^99^ bone scan showed hot spots over upper end of both humerii, trochanter of both femur, shaft of right femur (Figure 4). A bone marrow study showed 64% myeloperoxidase-negative blasts with scanty cytoplasm, blebbing, round nuclei and immature chromatin. The remaining cells in marrow showed a Myeloid, erythroid ratio of 1:2. Erythroid population showed dyserythropoiesis. Non erythroid population showed 4% blasts. Megakaryocytes were absent. These blasts were myeloperoixase negative and showed PAS block positivity. (Figure 5 and 6). Flow cytometry from marrow showed the blasts to be negative for CD13, CD33, CD64, CD117, cy MPO, cyCD61, CD10, CD19, CD2, CD3, CD4, CD5, CD5, CD8, cyCD3, CD34, and HLA DR. The blasts were positive for glycophorin A (Figure 7). Correlating the morphology, differential count and immunophenotype of blasts, a diagnosis of AML M6 (Pure erythroid leukaemia ) was made. The biopsy from the humerus shows spicules of bone with intervening neoplasm showing tumor cells in sheets (Figure 8). Cells were negative for LCA, MIC2 (Figure 9 and 10). The picture was compatible with AML M6 involving the bone. Bone marrow cytogenetics was normal, and Bcr Abl was negative. He was treated with induction chemotherapy with cytosine arabinoside and daunorubicin 7/3. He achieved remission and symptom relief from bone pain. He received further chemotherapy with FLAG for 3 cycles. His bone pain dissappeared and there were healing changes in the humerus. However, he relapsed 4 months later and was put on supportive care. He died of progressive disease at 10 months.

## Discussion

Although leukemia usually presents with pallor, bleeding tendencies, lymphadenopathy, and infections, rarely they present with skeletal manifestations. Such bone manifestations are more often found in lymphoid leukemias than myeloid. Osteolytic lesions of the skeleton associated with AML is uncommon. There are only few cases of AML associated with skeletal disease reported in literature (Table 1). Skeletal lesion has not been described in AML M6. The present case is the first report in literature of AML M6 presenting with skeletal lesions.

The radiological findings described in leukemias include metaphyseal lucent bands, bone erosions, periosteal reactions, lytic bone lesions, reduced bone density, permeative destruction and vertebral collapse.^9^ Bone lesions are more prevalent in children than in adults since growing skeleton is an important site for leukemic cell proliferation. Presence of bone lesions however do not give a worse outcome compared to those without bone involvement. Bone pain in acute leukemia is due to proliferation of bone marrow, pressure effect, compression fractures and osteoporosis.^10^ The pathogenesis of bone destruction in leukemia remain poorly defined. Abnormal production of parathyroid hormone by malignant cells has been demonstrated.^11^

The hematologic malignancies often presenting with osteolytic lesions are multiple myeloma, non Hodgkin’s lymphoma such as adult T cell lymphoma/leukemia, anaplastic large cell lymphoma. Bone involvement can also rarely occur in acute lymphoblastic leukemia and blast crisis of chronic myeloid leukemia.^12^ Other tumor presenting with predominant bone destruction at this age is Ewing’s sarcoma. In the present case, the bone was negative for LCA and MIC2, thus ruling out the possibility of a lymphoid malignancy and Ewing’s sarcoma.

The expression of glycophorin A on blast cells confirmed the diagnosis of erythroid leukemia. The present case demonstrates the importance of evaluation of skeleton in patients with AML presenting with bone pain.

## Figures and Tables

**Table 1:** Patients with AML presenting with bone involvement reported in literature

Reference	Age& Sex	Bony Sites involved	AML subtype	Outcome
Johnson JL 2	58 M	Multiple lytic lesions of L5, Skull, femur	AML M1	Relapsed at 10 months and died
Lima CS 3	17M	Loin pain and lytic lesion	AML	Relapsed after 12 months
Muler JH 4	32 M	Hypercalcemia and lytic lesion in skull, acetabulam, L1 and L5 vertebra	AML M7	Achieved remission and resolution of hypercalcemia
Fisher D 5	20 months F	Long bones, skull, jaw, short bones of hands	AML M7	Died 2 at 2 weeks
Franco A 6	8 months	Orbital wall fracture, periosteal reaction, mixed lytic and sclerotic lesion	MDS transformed to AML	NA
Dharmasena F 7	27M	Scapula, skull, pelvis, femur	AML M7	Relapsed 13 mths after diagnosis, underwent autologous transplant, died in posttranspalnt period
Seifis 8	21F	L3 vertebra, humerus	AML M2	Died
